# Pharmacokinetic and Metabolism Studies of Monomethyl Auristatin F via Liquid Chromatography-Quadrupole-Time-of-Flight Mass Spectrometry

**DOI:** 10.3390/molecules24152754

**Published:** 2019-07-29

**Authors:** Min-Ho Park, Byeong ill Lee, Jin-Ju Byeon, Seok-Ho Shin, Jangmi Choi, Yuri Park, Young G. Shin

**Affiliations:** College of Pharmacy and Institute of Drug Research and Development, Chungnam National University, Daejeon 34134, Korea

**Keywords:** MMAF, ADC, bioanalysis, metabolism, pharmacokinetics

## Abstract

A simple liquid chromatography–quadrupole-time-of-flight–mass spectrometric assay (LC-TOF-MS/MS) has been developed for the evaluation of metabolism and pharmacokinetic (PK) characteristics of monomethyl auristatin F (MMAF) in rat, which is being used as a payload for antibody-drug conjugates. LC-TOF-MS/MS method was qualified for the quantification of MMAF in rat plasma. The calibration curves were acceptable over the concentration range from 3.02 to 2200 ng/mL using quadratic regression. MMAF was stable in various conditions. There were no significant matrix effects between rat and other preclinical species. The PK studies showed that the bioavailability of MMAF was 0% with high clearance. Additionally, the metabolite profiling studies, in vitro/in vivo, were performed. Seven metabolites for MMAF were tentatively identified in liver microsome. The major metabolic pathway was demethylation, which was one of the metabolic pathways predicted by MedChem Designer. Therefore, these results will be helpful to understand the PK, catabolism, and metabolism behavior of MMAF comprehensively when developing antibody-drug conjugates (ADCs) in the future.

## 1. Introduction

An antibody-drug conjugate (ADC) is composed of an antibody, linkers, and payloads to deliver the cytotoxic payloads to target cells via antibody [1]. Four ADCs have been approved by US Food and Drug Administration (FDA) for the treatment of cancer; Mylotarg (gemtuzumab ozogamicin), Adcetris (brentuximab vedotin), Kadcyla (ado-trastuzumab emtansine), and Besponsa (inotuzumab ozogamicin) [2,3].

Payloads are mostly cytotoxic chemicals [4], such as maytansine [5], calicheamicin [6], auristatin [7], taxoid [8], etc. Among these chemicals, maytansine, auristatin, and calicheamicin are being used in FDA-approved ADCs. The auristatin is the most commonly used payload for ADCs and is currently in the clinical phase.

**Figure 1:**
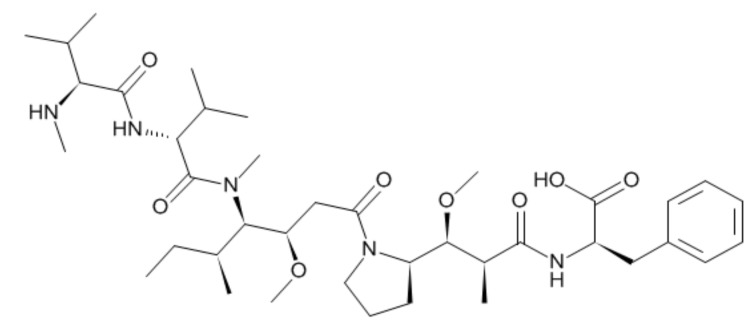
Structure of monomethyl auristatin F (MMAF).

Auristatin is a microtubule-destroying drug. It was derived from marine shell-less mollusk Dolabella auricularia called dolastatins (Figure 1) [9,10]. After the dolastatin was found, it has been studied in the treatment of cancer [11], malaria [12], and fungus [13]. After the successful total synthesis of dolastatin 10, various derivatives have been synthesized, such as monomethyl auristatin E (MMAE) and monomethyl auristatin F (MMAF) [14]. MMAE and MMAF were developed by Seattle Genetics and used as payloads for ADC. MMAF and MMAE have their advantages and disadvantages. MMAE is more membrane-permeable and has a lower IC50 than MMAF. However, MMAF is more hydrophilic and has a lower aggregation tendency to show lower systemic toxicity than MMAE [14,15,16,17,18].

Pharmacokinetic (PK) study is necessary for drug discovery and development process to help understand and predict the behavior of drugs and to link with the pharmacodynamics or toxicological effects of drugs [19,20]. Due to highly cytotoxic characteristics, PK studies of the payloads used for ADCs are important in understanding the behavior of the payloads [21,22]. Besides, a very sensitive assay is also required due to a very low dose of the payloads, and, therefore, routine analytical methods, such as LC-UV, etc., are not appropriate for the quantification of the payload drugs. There are a couple of studies in the literature related to auristatin-based drugs but are mostly related to synthetic methods [23,24], and only one analytical paper dealing with quantitation of dolastatin-10 has been reported so far [25]. There have been no reports related to pharmacokinetics, drug metabolism, metabolite identification of auristatin-based payloads, particularly MMAF, so far and, therefore, to our best knowledge, this paper is the first one dealing with the bioanalytical method development, in vitro/in vivo metabolite identification, and the qualification of MMAF in rat plasma.

The liver plays a major role in drug metabolism [26]. Liver microsome is generally used to understand drug metabolism in vitro. In this regard, in vitro-in vivo correlation has been proven in many publications [27,28,29]. It has also been studied that the ADC is degraded and excreted through the liver [30]. Therefore, in vitro/in vivo metabolism studies on payloads will be very helpful to understand and predict what happens to the released payload in the systemic circulation.

In this paper, a liquid chromatography–quadrupole-time-of-flight–mass spectrometric assay (LC-TOF-MS/MS) method for MMAF in rat plasma was developed and applied for the pharmacokinetic studies. Besides, metabolic profiling and identification studies were conducted for MMAF in vitro and in vivo. MMAF metabolites predicted by in silico were also compared with the metabolites observed by in vivo/in vitro studies. To our best knowledge, this is the first report for pharmacokinetics and metabolism of MMAF via LC-TOF-MS/MS.

## 2. Materials and Methods

### 2.1. Materials

MMAF was purchased from DC Chemical (Shanghai, China). Glutathione (GSH), uridine 5’-diphosphoglucuronic acid triammonium salt (UDPGA), and nicotinamide adenine dinucleotide phosphate reduced (NADPH) were purchased from Sigma-Aldrich (St. Louis, MO, USA). Dimethyl sulfoxide (DMSO), formic acid, and HPLC grade distilled water (DW) were purchased from Daejung Chemical (Gyonggi-do, Korea). Acetonitrile (ACN) was used of HPLC grade from JT Baker (Phillipsburg, NJ, USA). Rat liver microsome (RLM) and human liver microsome (HLM) were purchased from Corning (Tewksbury, MA, USA). All other chemicals were analytical or reagent grade and used without further purification.

### 2.2. Preparation of Stock Solution, Calibration Standard (STD), and Quality Control (QC) Samples

MMAF stock solution at a concentration of 1 mg/mL was prepared by dissolving 3 mg of MMAF in 3 mL of dimethyl sulfoxide (DMSO), and a sub-stock (0.1 mg/mL) was obtained by the dilution of stock solution. The working solutions for calibration standards (STDs) and QCs were then prepared by serially diluting the sub-stock using DMSO.

Seven calibration standards of MMAF were prepared in duplicate by spiking 20 μL of blank rat plasma with 4 μL of freshly prepared working standard solutions to achieve the final concentrations of 3.02, 9.05, 27.2, 81.5, 244, 733, and 2200 ng/mL, respectively.

Two levels of QCs were prepared by spiking 20 μL of blank rat plasma with 4 μL of QC solutions freshly prepared from the sub-stock solution of 0.1 mg/mL to obtain a final concentration of low QC (165.46 ng/mL) and high QC (1820 ng/mL).

### 2.3. Sample Preparation (Plasma Sample)

Twenty microliters of rat plasma samples were placed in a cluster tube. Four microliters (make-up solution) of DMSO were added to the cluster tube, and 100 μL of ACN was also added. The mixture was capped and vortexed, and then centrifuged for 5 min at 12,000 rpm (4 °C). Following the centrifugation, 70 μL supernatant was transferred to an LC vial and diluted by adding 140 μL of DW.

### 2.4. LC-TOF-MS/MS Conditions

LC-TOF-MS/MS was consisted of a Shimadzu CBM-20A/LC-20AD chromatographic system (Shimadzu Scientific Instruments, Columbia, MD, USA), with an eksigent CTC HTS PAL auto-sampler (Sciex, Redwood City, CA, USA) and a quadrupole time-of-flight TripleTOF 5600 mass spectrometer (Sciex, Redwood City, CA, USA). Kinetex XB-C18 column (2.1 × 50 mm, Phenomenex) for quantification and Kinetex XB-C18 column (2.1 × 100 mm, Phenomenex) for metabolite profiling studies were used as analytical columns. The LC mobile phase consisted of DW containing 0.1% formic acid (phase A) and ACN containing 0.1% formic acid (phase B). The LC-gradient is summarized in Table 1. The flow rate was 0.4 mL/min for quantification and 0.3 mL/min for Met ID while maintaining the column temperature at 55 °C. Injection volume was 10 μL.

(1) Quantification: For TOF-MS/MS scan, the scan range was *m/z* 100~900. The protonated [M + H]^+^ ion of MMAF (*m/z* 732.5) was used and its product ions at *m/z* 700.5 and *m/z* 520.3 were selected as the quantitative ion and the qualitative ion, respectively. The source temperature was set at 500 °C. The curtain gas flow was set at 30 L/min. The ion spray voltage (IVSF) was set at 5500 V. The declustering potential (DP) was 100 V, and the collision energy (CE) was 36 V.

(2) Metabolite profiling: The mass spectrometric conditions for the metabolite profiling of MMAF were as follows. The information-dependent analysis (IDA) with high-resolution TOF full scan was operated with electrospray ionization (ESI) in positive ion mode using real-time multiple mass defect filtering (MDF) and dynamic background subtraction. The IDA method consisted of TOF scan (*m/z* 50 to 1200) for the detection of MMAF and its metabolites. The method was followed by 6 TOF-MS/MS dependent scans (*m/z* 50 to 1200) for structural elucidation. The ion spray voltage (IVSF) was set at 5500 V. The source gas was settled at 50 psi, and source temperature was set at 500 °C with a curtain gas (CUR) flow of 33 L/min. The declustering potential (DP) was 100 V, and the collision energy (CE) was 10 V for TOF-MS scan and 35 V for TOF-MS/MS scan.

### 2.5. Method Qualification

The method development and qualification were performed with a ‘fit-for-purpose’ approach. The qualification run contained duplicate standards and QCs. The calibration curve was made with the quadratic regression function. Blank samples were also added. Sensitivity and selectivity were evaluated using the lower limit of quantification (LLOQ) and blank sample.

The species-dependent matrix effect on four other species (mouse, dog, monkey, and human) was assessed using QC samples. The samples were analyzed with the calibration curve prepared with rat plasma, and the accuracy/precision values were evaluated.

The extraction recovery of the protein precipitation was evaluated by comparing the concentration between extracted QC samples and post-extraction spiked QC samples. The extraction recovery was calculated by dividing extracted samples by post-extraction spiked sample.

The dilution integrity assessment was also performed on account of the possible PK samples showing higher concentrations above the upper limit of quantification (ULOQ), which may be diluted properly with blank rat plasma.

Preliminary stability tests were performed in rat plasma under 4 different conditions, including short-term, freeze-thaw, long-term, and post-preparative stability. The short-term stability was assessed at room temperature (RT) for 4 hrs. The long-term stability was assessed by comparing the freshly prepared control samples to the samples kept frozen for 4 weeks at −80 °C. For the freeze-thaw stability, the stability-test samples were subjected to three freeze and thaw cycles at −80 °C. The post-preparative stability was assessed for the QC samples kept in an auto-sampler (10 °C) for 12 hrs.

The acceptance criteria for all of the qualification were within ±15% of the precision and accuracy.

### 2.6. Software

Data acquisition and LC-MS/MS operation were conducted using Analyst® TF Version 1.6 (Sciex, Redwood City, CA, USA). MultiQuant® Version 2.1.1 (Sciex, Redwood City, CA, USA) was used for peak integration for MMAF quantification. The descriptive statistics for the qualification studies were calculated with Excel 2015 (Microsoft, Seoul, Korea). Pharmacokinetic parameters were calculated in a non-compartmental analysis using WinNonlin® version 8.0.0 (Certara, Princeton, NJ, USA). PeakView® Version 2.2 (Sciex, Redwood City, CA, USA) and MetabolitePilot^TM^ Version 2.0.2 (Sciex, Redwood City, CA, USA) were used for the structural elucidation of metabolites. MedChem Designer (Simulations Plus, Inc, Lancaster, CA, USA) was used for in silico metabolite prediction.

### 2.7. Application for a Pharmacokinetic Study in Rat

Male Sprague-Dawley (SD) rats were used for rat PK studies of MMAF. For rat PK studies of MMAF, rats were distributed into two different groups (5 mg/kg IV (intravenous) group and 10 mg/kg PO (oral) group). After IV or PO dose, blood samples were collected to heparinized tubes and centrifuged immediately at 10,000 rpm for 5 min. The sampling times were 0, 2, 5, 15, 30, 60, 90, 120, 240, 360, and 1440 min for IV PK and 0, 5, 15, 30, 60, 90, 120, 240, 360, and 1440 min for PO PK. The supernatant was transferred to a clean tube and stored at −80 °C until analysis.

### 2.8. Sample Preparation—In Vitro/In Vivo Metabolite Identification (Met ID)

(1) The enzymatic reaction of rat or human liver microsome was initiated by the addition of cofactors, such as NADPH (for oxidative phase 1 metabolism), UDPGA (for phase 2 glucuronidation), and GSH (for phase 2 glutathione conjugation) at a final concentration of 2, 5, and 0.5 mM, respectively. The cofactor-microsome mixture was pre-incubated at 37 °C for 3 min. The pre-incubated mixture was then transferred to Eppendorf tube, and MMAF was added to each tube. The incubation was carried out at 37 °C for 90 min. The reaction was stopped by adding ACN with protein precipitation. The quenched samples were gently vortexed and centrifuged at 12,000 rpm for 5 min, and the resulting supernatants were evaporated to dryness under vacuum in a rotary evaporator. The dried residue was re-constituted using DW/ACN (2:1), vortexed, centrifuged at 12,000 rpm for 5 min, and the supernatant was transferred to the LC-vial for analysis.

(2) Each of the in vivo samples from 10 mg/kg PO PK study and 5 mg/kg IV PK study was pooled per the Hamilton pooling method [31,32]. The pooled plasma samples were transferred to clean tubes, and ACN was added. After centrifugation at 12,000 rpm for 5 min, the resulting supernatants were evaporated to dryness under vacuum in a rotary evaporator. Dried residues were re-constituted, vortexed, and centrifuged similar to that in vitro metabolite profiling study. After the centrifugation was done, the supernatant was transferred to the LC-vial for analysis.

## 3. Results and Discussion

### 3.1. Method Development and Qualification

#### 3.1.1. Sample Preparation and Optimization of LC-TOF-MS/MS Parameters

**Figure 2:**
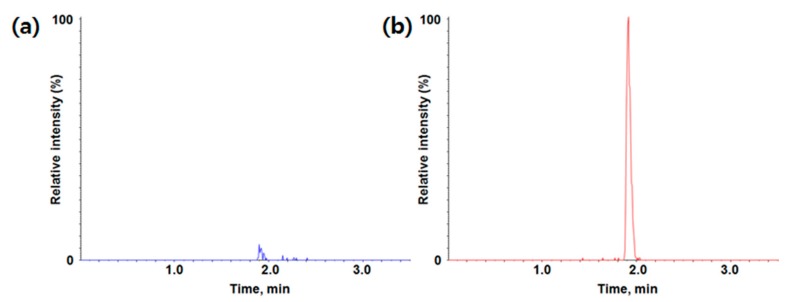
Typical chromatogram of (**a**) blank; (**b**) LLOQ (lower limit of quantification) for MMAF (monomethyl auristatin F) in rat plasma (*m/z* 732.5 -> *m/z* 700.5).

All sample preparations, as well as optimizations of LC-TOF-MS/MS parameters, were conducted from the ‘fit-for-purpose’ bioanalytical method validation perspectives [33,34,35]. Several conventional sample extraction methods, such as protein precipitation (PPT) [34], liquid-liquid extraction (LLE) [36], and solid-phase extraction (SPE) [37] techniques were also considered during method development, and the PPT method was selected for sample preparation in terms of simplicity and cost of the sample preparation. The PPT extracts were clean enough and met the sensitivity requirements without compromising accuracy and precision. There was no significant endogenous interference observed for MMAF using this sample preparation method (Figure 2).

LC-TOF-MS/MS in the positive ion mode was used for the quantification of MMAF (molecular weight 731.98 Da). The parent ion was the protonated [M + H]^+^ ion at *m/z* 732.5. The most abundant product ion was at *m/z* 700.5 in product ion scan mode, which appears to be a loss of CH_3_OH in MMAF.

Calibration curves in duplicate were prepared fresh for all data sets. The LLOQ of the assay was determined to be 3.02 ng/mL. Calibration range was 3.02~2200 ng/mL. Representative chromatograms of the blank matrix and LLOQ are also shown in Figure 2. A quadratic regression with an equation y = ax^2^ + bx + c was used to fit calibration curves over the concentration range for MMAF. The coefficient of correlation (r) value for calibration curves was used to evaluate the fitting of the curves, and the mean value was 0.9949.

#### 3.1.2. Method Qualification

Accuracy (%) and precision (% CV, coefficient of variation) of QC samples were evaluated for examining the assay performance, and the results are shown in Table 2. The qualification run met the acceptance criteria of ±15% accuracy and precision for all QC samples.

This developed assay for rat plasma samples was also evaluated for the plasma samples from other species (mouse, dog, monkey, and human). If there were no or little matrix effects between species, the rat plasma calibration curve could be applicable to quantitate MMAF in other preclinical species as well. The results showed that there were no significant species-dependent matrix effects between rat plasma and other species plasma (Table 3). Therefore, the rat plasma calibration curve may be able to analyze MMAF in mouse, dog, monkey, and human plasma samples, if needed.

The extraction recovery of MMAF was found to be 82.45 ± 2.12% (Table 4). The PPT method for sample preparation showed a robust recovery in MMAF.

If concentrations above the ULOQ are expected, the samples should be diluted with blank plasma, and the dilution integrity test should be conducted to confirm that the sample concentrations are within the calibration range and similar to the theoretical concentration after dilution. The accuracy (%) and precision (% CV) of the dilution QC samples (five-fold dilution using blank rat plasma) met the acceptance criteria of ±15% (Table 4). Therefore, PK samples with concentrations above ULOQ can be diluted up to five times with plasma.

The results for stability tests are summarized in Table 5 for MMAF. The accuracy (%) and precision (% CV) of stability samples met the acceptance criteria of ±15%. As a result, MMAF in rat plasma was stable under different experimental conditions (short-term, long-term, freeze/thaw, and post-preparative stability).

As seen above, LC-TOF-MS/MS method has been developed and qualified for the bioanalysis of MMAF in rat plasma. This qualified LC-TOF-MS/MS method enables PK study of MMAF in rats.

### 3.2. Application for Pharmacokinetic Study

**Figure 3:**
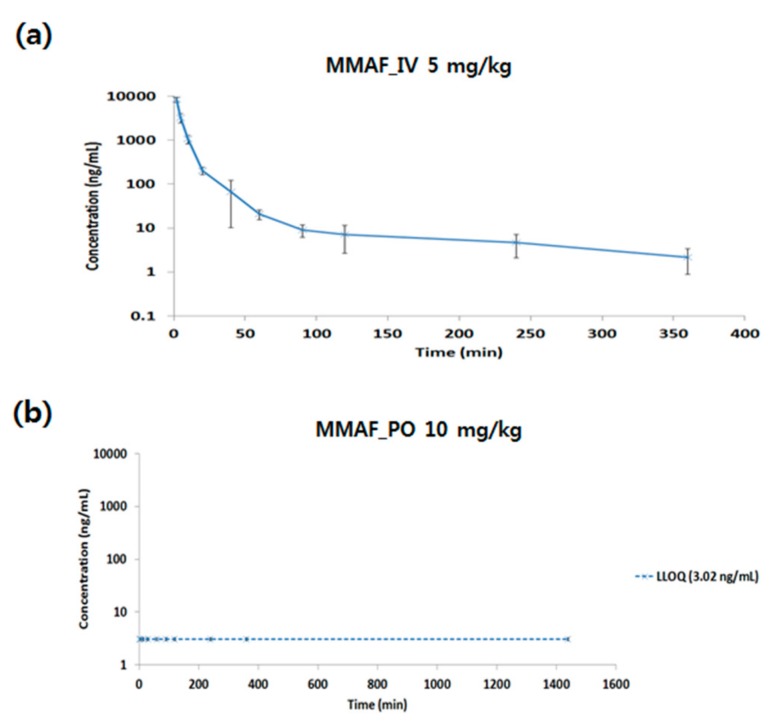
Pharmacokinetic profile of MMAF (monomethyl auristatin F) after (**a**) intravenous (IV) (5 mg/kg) or (**b**) oral (PO) (10 mg/kg) administration in rat.

The qualified LC-TOF-MS/MS method was successfully applied to the PK studies of MMAF in rats. In the PK studies of MMAF in rats, plasma samples obtained after intravenous (5 mg/kg) and oral administration (10 mg/kg) were analyzed by LC-TOF-MS/MS to quantify the concentration of MMAF. To assure acceptance of study sample analytical runs, at least two-thirds of the QC samples had to be within ±15% accuracy with at least half of the QC samples at each concentration meeting these criteria. Pharmacokinetic profile is shown in Figure 3.

The PK parameters are shown in Table 6. PK parameters were calculated using non-compartment model (NCA).

For IV PK of MMAF, Cmax was 8276.76 ng/mL. The AUClast was 65661.30 min*ng/mL, and the clearance (CL) was 77.33 mL/min/kg, which is above the hepatic blood flow in the rat. The volume of distribution (Vss) was 1057.13 mL/kg, which is above the blood volume in rat. Based on this information, extra-hepatic clearance also plays a role in terms of MMAF CL, and MMAF might be distributed not only in blood but also in tissues. Also, for PO PK of MMAF, no systemic exposure was observed. The reason for the low bioavailability (BA) might be due to low permeability, solubility, or high clearance in vivo.

### 3.3. In Vitro and In Vivo Metabolite Profiling for MMAF

**Figure 4:**
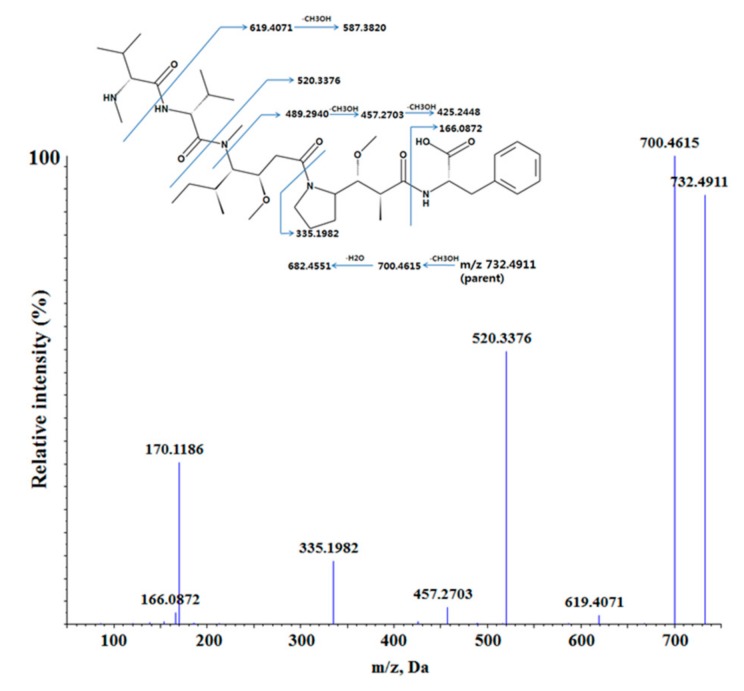
The proposed fragmentation pattern of MMAF (monomethyl auristatin F) in positive ion mode.

Metabolite profiling study of MMAF was performed in vitro using rat and human liver microsomes fortified with NADPH, UDPGA, and GSH. MMAF had a molecular ion of *m/z* 732.4911 with the retention time of 25.1 min. The product ion scan of *m/z* 732.4911 led to the formation of fragment ions at *m/z* 700.4615, 682.4551, 619.4071, 587.3820, 520.3376, 489.2940, 457.2703, 425.2448, 335.1982, and 166.0872 (Figure 4). The peak intensities of some fragment ions, such as 682.4551, 587.3820, 489.2940, 425.2448, and 166.0872, were quite low and not shown in Figure 4. However, these ions were useful and were included in the list when compared with the fragment patterns of the metabolites of MMAF for the elucidation of metabolites.

**Figure 5:**
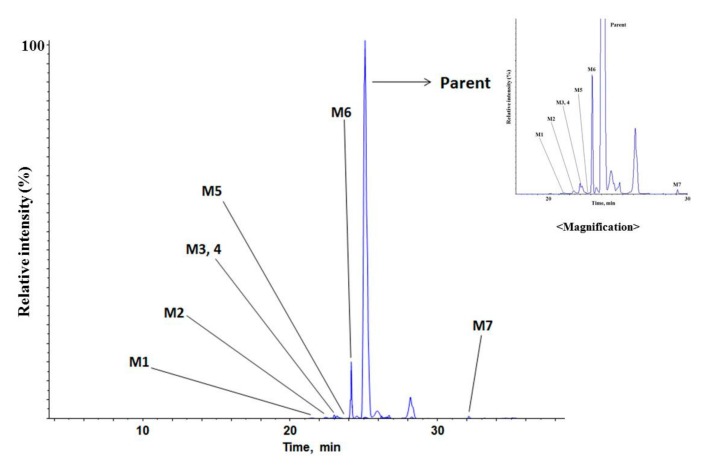
Total ion chromatogram of MMAF (monomethyl auristatin F) and its metabolites in human liver microsome.

Total ion chromatogram (TIC) of the parent and the proposed metabolites of MMAF in human liver microsome are presented in Figure 5.

**Figure 6:**
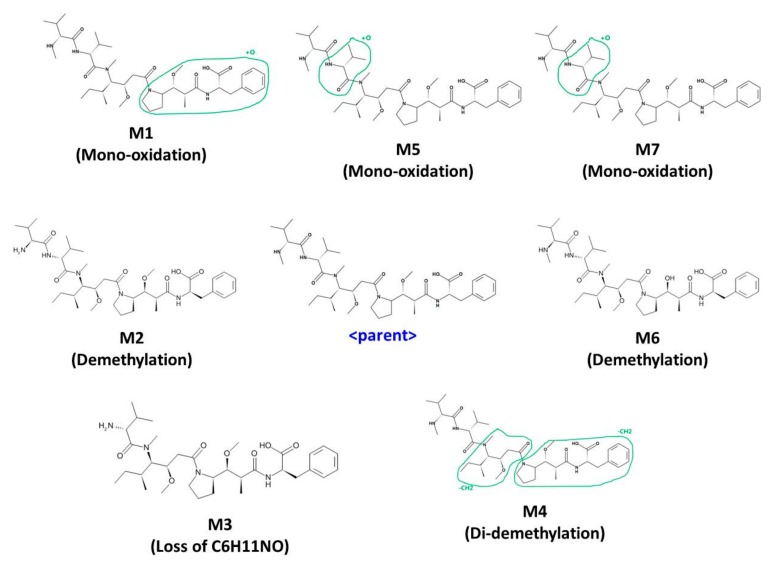
Metabolic pathway of MMAF (monomethyl auristatin F); Mono-oxidation, Demethylation, Di-demethylation, Loss of C6H11NO.

Under the current experimental conditions, seven metabolites were tentatively identified in human liver microsome. The metabolic pathways are shown in Figure 6. The detected and identified metabolites are shown in Table 7.

There were four kinds of metabolites of MMAF. They are characterized by mono-oxidation, demethylation, di-demethylation, loss of C6H11NO. Each of the characterized metabolites had various sites of metabolism (Figure 6). M5 and M7 were suggested to be mono-oxidative metabolites. However, due to the similarity of the fragment pattern, it was difficult to find the exact metabolic site of oxidation. Oxidated metabolites usually are more hydrophilic than parent and are eluted before parent during the reverse-phase LC analysis. However, M7 was eluted after parent. It is usually N-oxide-type metabolites that are eluted at a later time than the parent when oxidation occurs [38,39,40]. Therefore, M5 was presumed to be oxidized to dimethyl groups within the range suggested, whereas M7 was presumed to be oxidized to the nitrogen or more sterically hindered location in the structure. Among the metabolites detected in the current incubation condition, M6 (de-methylated MMAF) was the most abundant based on its peak intensity. These results suggest that the major metabolic pathway of MMAF is demethylation.

Under the current experimental conditions, four metabolites and seven metabolites were tentatively identified in rat and human liver microsomes, respectively. This phenomenon would be due to the difference in metabolic enzymes between rat and human.

Before the actual experiment, metabolites for drugs were also predicted using a program called MedChem Designer. Three mono-oxidations and one de-methylation by CYP3A4 were predicted, totaling four metabolites. These results suggested that there was a correlation between in silico and in vitro/in vivo results, and the in silico tool was very useful to predict in vitro/in vivo metabolites.

Metabolite profiling and identification were performed using pooled rat plasma dosed at 5 mg/kg (IV) or 10 mg/kg (PO). Each of the in vivo samples was pooled per the Hamilton pooling method [31]. Under the current experimental conditions, the metabolites identified from the liver microsomes in vitro were not found from the in vivo rat plasma samples (Table 7). Also, significant levels of novel metabolites were not detected from the rat plasma samples, either. This result implies that the in vivo high clearance of MMAF in the rat would be likely due to excretion-mediated elimination rather than metabolism-mediated elimination.

## 4. Conclusions

LC-TOF-MS/MS method was developed and qualified for the quantification of MMAF in rat plasma. The calibration curves were acceptable over the concentration range from 3.02 to 2200 ng/mL using quadratic regression. This LC-TOF-MS/MS method was selective, accurate, and reproducible for the determination of MMAF concentration. The stability of MMAF in various conditions was also acceptable. There were no significant matrix effects between rat and other preclinical species (mouse, monkey, dog, and human), which suggests these methods would be easily applicable for other preclinical sample analysis for MMAF. These methods have been successfully applied for the bioanalysis of MMAF in rat plasma samples. The PK results suggested that MMAF had very high CL and low exposure, and this phenomenon was not likely due to metabolism-mediated elimination of MMAF based on the in vitro and in vivo metabolite profiling and identification results.

In conclusion, these results will be helpful to understand the PK, catabolism, and metabolism behavior of MMAF comprehensively when developing ADCs in the future.

## Figures and Tables

**Table 1:** Mobile phase conditions for LC gradient.

Quantification of MMAF	
Time (min)	Mobile phase B (%)
0	10
0.5	10
1.3	95
1.9	95
2.0	10
3.5	10
**Metabolite profiling of MMAF**	
**Time (min)**	**Mobile phase B (%)**
0	5
2	5
28	33
29	33
34	95
38	95
38.1	5
45	5

**Table 2:** Quality control (QC) results for MMAF (monomethyl auristatin F) in rat plasma.

	Low QC (165.46 ng/mL)	High QC (1820 ng/mL)
Mean concentration (ng/mL)	163.94	1832.78
Accuracy %	99.08	100.70
% CV	2.52	2.83
n	10	10

**Table 3:** The species-dependent matrix effect of MMAF (monomethyl auristatin F).

	Low QC (165.46 ng/mL)				
Species	Mouse	Rat	Dog	Monkey	Human
Mean concentration (ng/mL)	184.60	155.72	171.41	171.36	164.36
Accuracy %	111.57	94.11	103.60	103.57	99.34
% CV	6.36	4.03	2.97	6.59	7.16
n	3	3	3	3	3
	**High** **QC (1820 ng/mL)**				
**Species**	**Mouse**	**Rat**	**Dog**	**Monkey**	**Human**
Mean concentration (ng/mL)	1651.07	1792.99	1796.06	1667.92	1873.39
Accuracy %	90.72	98.52	98.69	91.64	102.93
%CV	7.51	6.08	3.52	6.14	2
n	3	3	3	3	3

**Table 4:** Extraction recovery and dilution integrity results of MMAF (monomethyl auristatin F) in rat plasma.

MMAF	QC
Mean concentration of the post-extraction spiked QC (ng/mL)	198.84
Mean concentration of the extracted QC samples (ng/mL)	163.94
Extraction recovery (%)	82.45
% CV	2.12
n	3
**MMAF**	**Dilution QC (9100 ng/mL)**
Mean concentration (ng/mL)	8091.72
Accuracy %	88.92
% CV	9.02
n	3

**Table 5:** Stability assessments of MMAF (monomethyl auristatin F) in rat plasma (a) Short-term stability; (b) Long-term stability; (c) Freeze-thaw stability; (d) Post-preparative stability.

(a) Short-term stability (RT, 4 h)	Low QC (165.46 ng/mL)			High QC (1820 ng/mL)		
Incubation time (hr)	0 (Control)	4		0 (Control)		4
Mean Concentration (ng/mL)	169.87	149.90		1970.02		1884.23
Accuracy %	102.67	90.60		108.24		103.53
% CV	2.08	3.05		5.20		3.27
n	3	3		3		3
(b) Long-term stability (−80 °C, 4 weeks)	Low QC (165.46 ng/mL)			High QC (1820 ng/mL)		
Mean concentration (ng/mL)	157.95			1657.96		
Accuracy %	95.46			91.10		
% CV	8.32			11.93		
n	3			3		
(c) Freeze-thaw stability (−80 °C, three cycles)	Low QC (165.46 ng/mL)			High QC (1820 ng/mL)		
Mean concentration (ng/mL)	157.95			1657.96		
Accuracy %	95.46			91.10		
% CV	8.32			11.93		
n	3			3		
(d) Post-preparative stability (10 °C, 12 h)	Area ratio of 1st injection		Area ratio of 10th injection (after 12hr)	Change (%)	% CV	
Low QC	0.62		0.61	97.93	2.67	
High QC	7.43		7.43	99.98	0.43	

**Table 6:** Pharmacokinetic parameters of MMAF (monomethyl auristatin F) after intravenous/oral administration in rats.

Subject	Dose (mg/kg)	Cmax (ng/mL)	AUClast (min*ng/mL)	Clearance (CL) (mL/min/kg)	Vss (mL/kg)	Bioavailability (%)
MMAF IV (5 mg/kg)	5	8276.76	65661.30	77.33	1057.13	
MMAF PO (10 mg/kg)	10	N/D	N/D			0

**Table 7:** Characterization of MMAF (monomethyl auristatin F) and its metabolites (error ppm ≤5 ppm).

Symbol	Metabolite	*m/z*	Retention Time (min)	Rat Liver Microsome	Human Liver Microsome	Pooled Rat Plasma (IV)	Pooled Rat Plasma (PO)
M1	Oxidation-1	748.4624	21.50		0	N/D	N/D
M2	Demethylation-1	718.4519	22.40	0	0	N/D	N/D
M3	Di-demethylation	704.4362	22.96		0	N/D	N/D
M4	Loss of C6H11NO	619.3813	23.17	0	0	N/D	N/D
M5	Oxidation-2	748.4624	24.01		0	N/D	N/D
M6	Demethylation-2	718.4519	24.55	0	0	N/D	N/D
Parent	Parent	732.4911	25.10	0	0	0	0
M7	Oxidation-3	748.4624	32.16	0	0	N/D	N/D

N/D = not detected.
